# Influence of the sociocultural perspective on the sensory perception of wine consumers in Mexico and Spain

**DOI:** 10.3389/fpsyg.2023.1171289

**Published:** 2023-06-09

**Authors:** Elena Sánchez, Ingrid Oliveras, Maria Roser Romero del Castillo, Angeles Salazar

**Affiliations:** Departament d’Enginyeria Agroalimentària i Biotecnologia, Universitat Politècnica de Catalunya, UPC, Castelldefels, Spain

**Keywords:** wine, sociocultural perspectives, cross-cultural study, consumer perception, sensory analysis, Spain, México

## Abstract

The increasing globalization of companies and markets, including the wine market, makes this study important as a cultural comparison between the sensory perception of wine in Mexico and Spain. Eighty consumers were selected with different consumption habits, and hedonic (Acceptance and Simple Preference) and descriptive (Word Association Task and the Check-All-That-Apply (CATA) method) sensory tests were performed. The results revealed that there were differences in the conceptualization of wine in the Word Association Task. Both populations preferred wines of Spanish origin over those of Mexican origin, especially in the case of red wine. Finally, the results of the CATA method showed that the attributes that discriminate the two types of wine are due more to the country origin of the tasters than to the samples. Spanish consumers used cultural and tradition descriptors and were stricter when it came to sensory evaluation. Moreover, Spanish participants demonstrated more ability to differentiating all wines in terms of visual, olfactory and taste aspects.

## 1. Introduction

For centuries, the human race has inhabited all corners of the world as groups of people that, by working and living together, have developed the different cultures that we have today (UNESCO, 2012). The interaction between two or more cultures and the possibility of generating shared expressions in an equal way is known as interculturality (UNESCO, 2005). The main emphasis of cross-cultural research is the identification of similarities and differences in behaviors or concepts across cultures. The assessment of commonalities enables the comparison and measurement of participants from diverse cultures and backgrounds within this globalized society (Holgado-Tello et al., 2022).

In recent decades, cross-cultural perception and preferences studies regarding food sensory attributes have been developed and intercultural studies are increasingly relevant in the sensory and consumer sciences (Ares, 2018). Some of these studies have focused on explaining differences in food selection in different cultures based on basic tastes or other sensory qualities (Wan et al., 2016; Sorokowska et al., 2017; Cecchini et al., 2019). Other studies are focused on marketing strategies for the development of business models in other countries different from where the evaluated products come from, such as, for example, nopal (De Albuquerque et al., 2019), cider (Jamir et al., 2019), balsamic vinegar (Torri et al., 2017), and moringa leaf (Rakotosamimanana et al., 2015).

In recent years, the wine industry and the research community have begun to apply principles of sensory evaluation to quantify consumer preferences, such as studies of perceptions of naturalness (Staub et al., 2019), oxidation (Franco-Luesma et al., 2019), minerality (Parr et al., 2015), astringency, and the benefits consumers associate with the product (Yoo et al., 2013; Chang et al., 2016). There are also other studies on wine in reference to the contribution of psychology in the demystification of wine tasting (Parr, 2019), the contribution of psychology in the demystification of wine tasting (Parr, 2019), the contribution of the studies (Rodrigues and Parr, 2019), the emotional response (Mora et al., 2018), the application of consumer sensory science (Francis and Williamson, 2015), extrinsic attributes (Sáenz-Navajas et al., 2014) and intrinsic quality-related attributes (Sáenz-Navajas et al., 2013). On the other hand, there is also research on the motives underlying purchase decisions (Ginon et al., 2014; Fiore et al., 2017; Galati et al., 2019; Salvatore et al., 2021), and innovative perspectives, such as the use of blockchain technology such as the use of blockchain technology or other models and simulators (Adamashvili et al., 2021).

According to Parr et al. (2011), there are several variables that interact with culture to influence the appreciation of a food, especially domain-specific experience. The level of familiarity with the food product could have a strong impact on product description by subjects from different cultures (Torri et al., 2017). Similarly, the use of descriptors in different continents is influenced by being more exposed to the type of product analyzed by sensory analysis methods and according to each culture (Jamir et al., 2019). A conclusion in these studies is that there are several factors associated with food behavior, such as economic, biological, and individual issues, despite the fact that the perception of food qualities and basic flavors are the same for everyone the first time they perceive a new taste (Cecchini et al., 2019). Therefore, from the industrial point of view, conducting sensory research provides information to help producers create a product that is closer to the tastes of each population and, thus, adapt them for proper acceptance (De Albuquerque et al., 2019).

The wine sector is of extraordinary relevance in Spain, not only from an economic or environmental point of view, but also from a social and cultural aspect. In 2018, a total of 969,000 Ha were cultivated in Spain, which represents approximately 13% of the world’s total wine-producing area, making it the country with the largest vineyard area in the world. On the other hand, the Mexican wine market is in a growing phase, both in consumption and available variety, and has undergone profound changes in the last few years (Consejo Mexicano Vitivinícola, 2018). According to data from the International Wine Organization (OIV, 2021), the area of vines planted in 2018 was 37.000 Ha, which represents almost 0.5% of the world area. The difference between production and consumption is noticeable in traditional wine-producing countries, for example, production in Spain in 2021 was 35 million hectoliters while consumption was 11 million hl, which means an average of 27.8 liters *per capita* per year (OIV, 2021). However, the production in Mexico was 395 thousand hectoliters and the consumption was less than one liter *per capita* per year (Consejo Mexicano Vitivinícola, 2018). Given the differences in the wine world between the two countries, it is interesting to conduct a cross-cultural study reflecting the preferences and sensory perceptions of red and white wines produced in both places, thus achieving a better understanding of the sensory experiences that are related to culture and expertise or exposure to the product.

Wine tasting is the procedure for appreciating the qualities of a wine through the senses of sight, smell, taste, and mouthfeel (Lawless and Heymann, 2010). Tasting is subjecting a wine to the senses in order to try to get to know it and determine its characteristics, and finally to appreciate it. According to Stone and Sidel (2004), wine is made to be consumed and appreciated. The sensory analysis of wines consists of describing them in terms of the organoleptic properties that characterize them. This analysis makes it possible to evaluate the different types of wine as well as to appreciate certain nuances of certain characteristics within the tasting (Stone and Sidel, 2004). In addition, wine is a traditional product closely linked in the Mediterranean area to social relations, starting with the family and continuing with friends. Wine is a coexistence symbol that is present in all important meals, influencing the sensory memory of the consumers. Understanding how people consume wine in other cultures where wine consumption is relatively recent is an interesting approach to discover how their sensory memory is built in relation to this product and how to direct consumers towards the consumption of products with a higher sensory quality and in which circumstances to consume it.

The general objective of this study is to determine the perception and preference of red and white wines in the Mexican and Spanish populations, using sensory tests for each case. Moreover, the specific objectives of the research are to establish the sensory differences of the target beverage between both populations by means of quantitative (acceptance test, CATA, and simple preference) and qualitative (word association task) sensory methods, as well as to determine the preference of the panelists for the wines presented in the panel test.

## 2. Materials and methods

### 2.1. Wine and participants

Four commercially available wines (two whites and two reds) from Spain and Mexico were chosen in this cross-cultural study. Extrinsic criteria were established for their selection: variety, single varietal, vintage, alcohol content, aging time, type of aging, and price (Table 1). The selection of varieties was based on those produced in both countries. The L.A. Cetto winery in Valle de Guadalupe in the state of Baja California, Mexico, was chosen as it is in one of the main wine producing areas in Mexico. Additionally, a Spanish winery that exports wine to Mexico and produces wines with same varieties was selected. Therefore, the Enate winery (D.O. Sotomontano) was the one that was best suited to the requirements. A total of 16 bottles were used, four bottles of each type of wine (white or red) and origin (Mexico or Spain). The wine samples were randomly coded with three digits (Price et al., 2019): 731 (Enate white wine), 649 (L.A. Cetto white wine), 410 (Enate red wine), and 218 (L.A Cetto red wine). The wine was served according to the assigned code, pouring approximately 0.075 liters of each sample. The wines were previously refrigerated at a temperature of 8°C for the whites and 15°C for the reds, so that their aroma and flavor could be properly appreciated.

**Table 1:** Technical specifications of the wines used for the research.

Wine name	L.A. Cetto Cabernet Sauvignon	Reserva Cabernet Sauvignon	L.A. Cetto Chardonnay	Chardonnay 234
Winery name	Vinícola L.A. Cetto	Enate	Vinícola L.A. Cetto	Enate
Type of wine	Red wine	Red wine	White wine	White wine
Grape variety	Cabernet Sauvignon	Cabernet Sauvignon	Chardonnay	Chardonnay
Wine area	Valle de Guadalupe, Baja California, México	D.O. Somontano, Aragón, España	Valle de Guadalupe, Baja California, México	D.O Somontano, Aragón, España
Harvest	2015	2012	2017	2017
ºAlcohol	13.5%	14.5%	13%	14%
Time and type of ageing	12 months Oak barrel	12 months Oak barrel	–	–

For the present study, semi-trained judges were the most suitable one because of their sensory acuity and previous preparation and participation in simple discriminative tests (Anzaldúa-Morales, 2005). In this case, students and professors from the Bachelor’s Degree in Gastronomy of the Universidad del Claustro de Sor Juana (now UCSJ) in México and from the Culinary and Gastronomic Sciences Degree, Higher Technician in Restaurant Services, and other CETT-UB training courses in Spain were considered. Those selected participants closely matched the desired profile because they had studied subjects related to the world of wine (oenology and sommelier) and sensory analysis. Therefore, they were able to discriminate the products and interact with them without the need for training prior to sensory evaluation. A total of 80 participants were selected (49 men and 31 women), 40 from each population.

### 2.2. Sensory evaluation

Once the wines were selected and the sensory panel was formed, sensory evaluations were implemented at sensory classrooms of the universities participating for data collection: Universidad del Claustro de Sor Juana in Mexico City and Universitat de Barcelona in Barcelona. In this study, four analysis tests were carried out: three quantitative test (the acceptance test, CATA, and simple preference test) and one qualitative test (the word association task). The four tests were presented to the panelists on a single sensory card, which asked for demographic data (age and gender) and included the structure of the sensory evaluation, indicating the samples to be evaluated and the four tests to be performed, about which they were asked to read the instructions carefully.

### 2.3. Quantitative tests

#### 2.3.1. Acceptance test

For the wine acceptance test, a 9-point hedonic scale (Pagliarini et al., 2013) was presented where 9 meant “*I like it very much*,” 5 meant “*I neither like nor dislike it*,” and 1 meant “*I dislike it very much*.”

For the statistical analysis, R statistical program^1^ was used. Analysis of variance (ANOVA) was performed on four factors: sample, country, gender, and country-gender interaction. To find significant differences, a significance of *p* < 0.05 was considered between the means of the data in white wine and red wine. In addition, Tukey’s test was performed to see the honestly significant difference (HSD). This is a test used to find out if the means of acceptance of white wine and red wine are significantly different, as well as the means of acceptance of wine according to country.

#### 2.3.2. CATA method

In the CATA (Check-All-That-Apply) method, tasters were asked to mark all the attributes that best describe the four samples, based on visual, olfactory, and gustatory aspects. Within each aspect, they had to choose between different attributes proposed for the description of the samples (wine), marking the terms they considered that best matched their sensory perception. The selected attributes were the most common to a wine tasting sheet: for the visual attributes, attributes related to color, limpidity, and color intensity were chosen; for the olfactory attributes, aromatic intensity and complexity; and finally, for gustatory attributes, attributes such as alcohol content, bitterness, astringency, acidity, and persistence. However, some olfactory attributes were previously selected through a tasting session, selecting the detected aromas specific to each wine. With the results obtained from the CATA method, a matrix of 0 and 1 was made with which a contingency table was made for each wine (Hiscock et al., 2020). Each of the values corresponds to the sum of the number of times a given attribute was selected for each of the two samples of each type of wine according to country.

A statistical analysis was then performed with XLSTAT (2019) software using Cochran’s Q test considering probability of *p* ≤ 0.05 to determine whether or not there were significant differences between products for each of the attributes. To find out where the significance lies, a new statistical analysis was performed with XLSTAT, according to the Sheskin procedure of multiple paired comparisons, to determine, at a confidence level of 95%, between the differences for each of the attributes that were discriminatory.

#### 2.3.3. Simple preference test

In the simple preference test, the tasters were instructed to choose which sample of each wine they preferred. To facilitate the interpretation of the data obtained, the binomial distribution was used as a statistical method to determine whether the outcome of the study was due to chance or whether the panelists preferred one wine over the other (Lawless and Heymann, 2010), where the probability of success of choosing between the two samples of each wine is 50%. Furthermore, for the probability of preference (P) to be significant between samples (<0.05), there must be a minimum of 26 responses from 40 participants from each country and 48 from 80 participants in both countries (Roessler et al., 1978). However, for the analysis, the probability must be <0.025 to be significant, since it is distributed between the two tails of the Gaussian curve because we do not know *a priori* which wines will be preferred.

### 2.4. Qualitative tests

#### 2.4.1. Word association task

In the word association task, the tasters were asked through an open-ended question what wine meant to them. All valid phrases and words mentioned by the participants were considered in the data analysis. For the analysis, the terms mentioned by the participants were classified into dimensions and categories for each country (De Albuquerque et al., 2019). For the classification, a process of normalization by lemmatization (i.e., Tasting and taste) and paraphrase grouping (i.e., understanding and knowledge) of the phrases and words used to mention wine was carried out. Those that were mentioned only once or could not comply with the normalization were annulled. Thereupon, an enumeration was made of the categories in each country based on the terms mentioned. For example, if six terms were mentioned in the “Sensory Aspects” category, this category was given a value of 6.

## 3. Results and discussion

### 3.1. Sample description

In terms of demographic data, both populations showed a higher number of men (49) than women (31). The Mexican population was made up of 67.5% men and 32.5% women, while the Spanish population was slightly more equal, with 55% of men and 45% of women. With regard to the ages of the participants, the age range was between 18 and 65 years, with the most frequented age range being 18 to 25 years in both countries. However, the demographic data were not considered for the results, as the data analysis did not find any significant differences between the populations in the data analysis.

### 3.2. Quantitative tests

#### 3.2.1. Acceptance test

The analysis of variance (ANOVA) of the means of the white and red wine data are shown in Table 2. In the case of white wine, significant differences were only observed between samples, whereas red wine showed significant differences both between samples and between countries.

**Table 2:** ANOVA results for white and red wine as a function of sample, country, gender, and country-gender interaction.

		*F*	Significance
White wine	Sample	7.582	0.0066^**^
	Country	0.842	0.3501
	Gender	1.562	0.2132
	Country^*^Gender	3.454	0.0650
Red wine	Sample	9.119	0.0029^**^
	Country	18.094	3.63·10^−5***^
	Gender	1.770	0.1853
	Country^*^Gender	1.121	0.2913

*F*: radius of variability, value equal to 1, the farther away, the greater the significant difference. ^*^ means *p* < 0.05; ^**^ means *p* < 0.01; ^***^ means *p* < 0.001. Honestly significant differences (HSD).

After applying Tukey’s test, participants showed a greater acceptance for the Enate white wine (6.5) than the L.A. Cetto white wine (5.8), and similar was observed with the red wine, where the Enate wine had greater acceptance than the L.A. Cetto (5.8 and 5.0, respectively). Although with not very high marks, all the wines were approved by both populations. The white wine showed a similar acceptance for the participants according to country (6.3 and 6 in Mexico and Spain, respectively) and non-significant differences were found. However, significant differences were found in the red wine between countries (5.9 and 4.8 in Mexico and Spain, respectively).

The greater preference for Spanish wines (Table 2) could be due to the fact that both populations (Mexico and Spain) are accustomed or habituated to consuming Spanish wines. Habits are behaviors that we repeat many times until they become part of our daily activities. Reeves and Baden (2000) define habits as the distinctive element of ideas, beliefs, and norms which characterize the way of life and relations of a society or group within a society. The fact that Spanish wine consumption accounts for 30% of national consumption in Mexico (Consejo Mexicano Vitivinícola, 2018) means that Mexicans consumers are habituated to Spanish wines, and therefore prefer them to those of their own country, which would be expected.

#### 3.2.2. CATA method

The results of Cochran’s Q test (Varela and Ares, 2012) from the contingency tables for each wine determined that 12 out of the 31 attributes presented had significant differences (*p* < 0.05) in the white wine, and were “*White*,” “*Pale yellow*,” “*Golden yellow*,” “*Very bright*,” “*Not very bright*,” “*Very aromatic*,” “*Not very aromatic*,” *“Low aromatic*,” “*Simple complexity*,” “*Medium complexity*,” “*Complex*,” “*Green apple*,” and “*Persistent in the mouth*.” On the other hand, seven out of the 33 attributes presented had significant differences (p < 0.05) in the red wine, and were “*Ruby red*,” *“Cloudy*,” “*Very bright*,” “*Not very bright*,” “*Woody aromas*,” “*Vegetables aromas*,” and “*Very alcoholic*.” In both cases, the attributes that did not discriminate wines represented more than 60% of the total.

After performing a second analysis using Sheskin’s paired comparison to determine between which samples there were significant differences, the attributes that discriminated the wines were due more to the country than to the samples. In both cases, Spanish participants significantly differentiated more wine attributes than Mexicans participants. This could be due to a greater knowledge of sensory aspects of wines by the Spanish participants, following the higher wine consumption by the Spanish population compared with the Mexican population (OIV, 2021).

**Figure 1:**
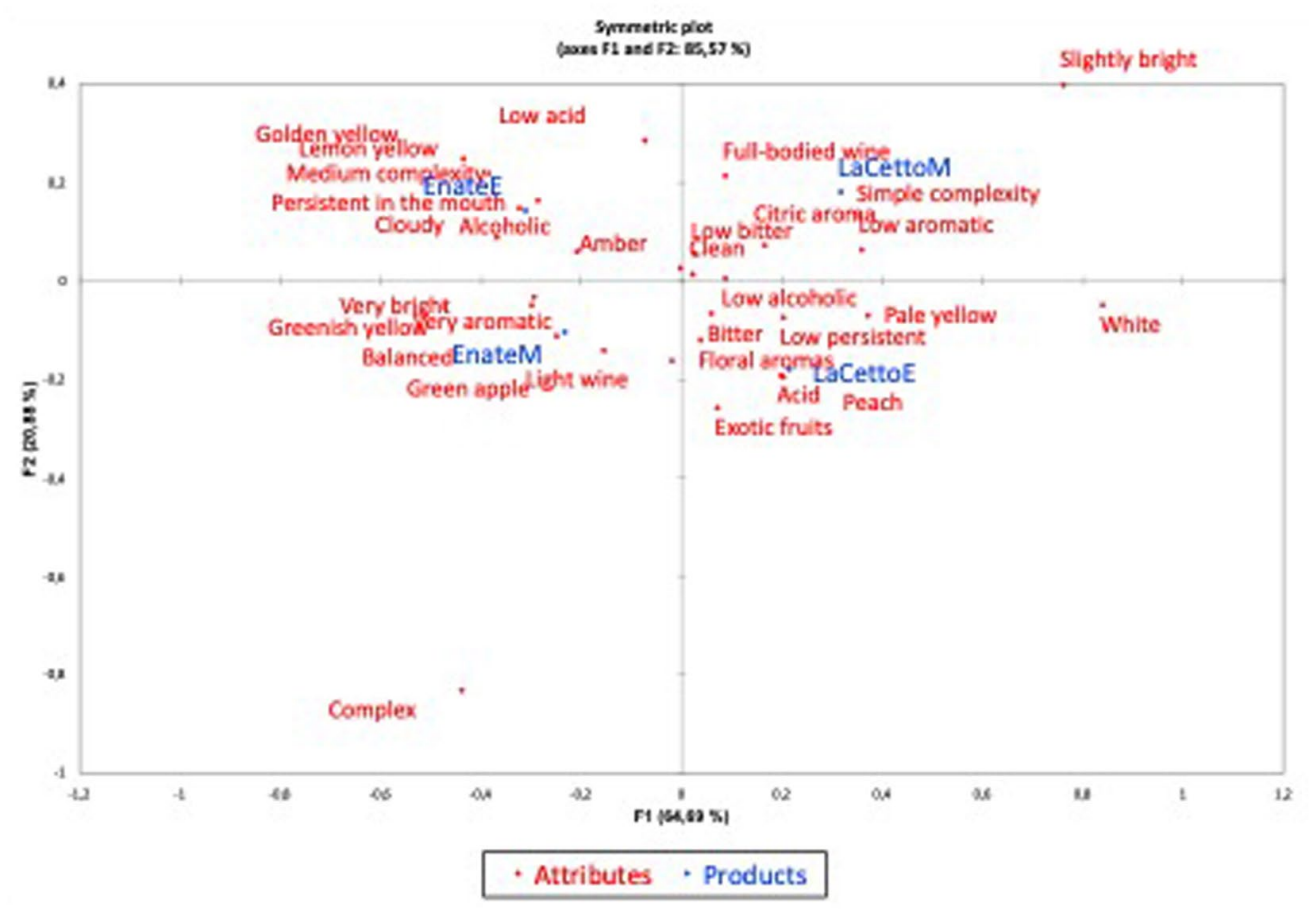
Principal coordinate analysis of white wine attributes and products. Note: F: Dimensions. Attributes: white wine attributes. Products: wine sample by country. EnateM (Enate wine tested in Mexico), EnateE (Enate wine tested in Spain), LaCettoM (L.A. Cetto wine tested in Mexico), LaCETTOE (L.A. Cetto wine tested in Spain).

The first and second dimensions (F1 and F2) of the multiple correspondence analysis (MCA) for the white wine (Figure 1) accounted for 85.57% of the variance among the wines, representing 64.69 and 20.88% of the variance, respectively (Parente et al., 2011). This high percentage of variability indicates that the attributes used by both populations (Mexican and Spanish) have characterized each wine differently, placing the wines in the graph close to the words that most describe them.

The Enate white wine was perceived by the Spanish participants as “*Medium complexity*,” “*Persistent in the mouth*,” and “*Alcoholic*,” while according to the perception of the Mexican participants it was “*Balanced*,” “*Very bright*,” and *“Light wine*.” On the other hand, the L.A. Cetto white wine was perceived by Mexican participants as “*Full-bodied wine*,” “*Simple complexity*,” and “*Not very aromatic*,” while Spanish participants characterized it using “*Floral aromas*,” “*Exotic fruits*,” and “*Bitter*,” among others.

**Figure 2:**
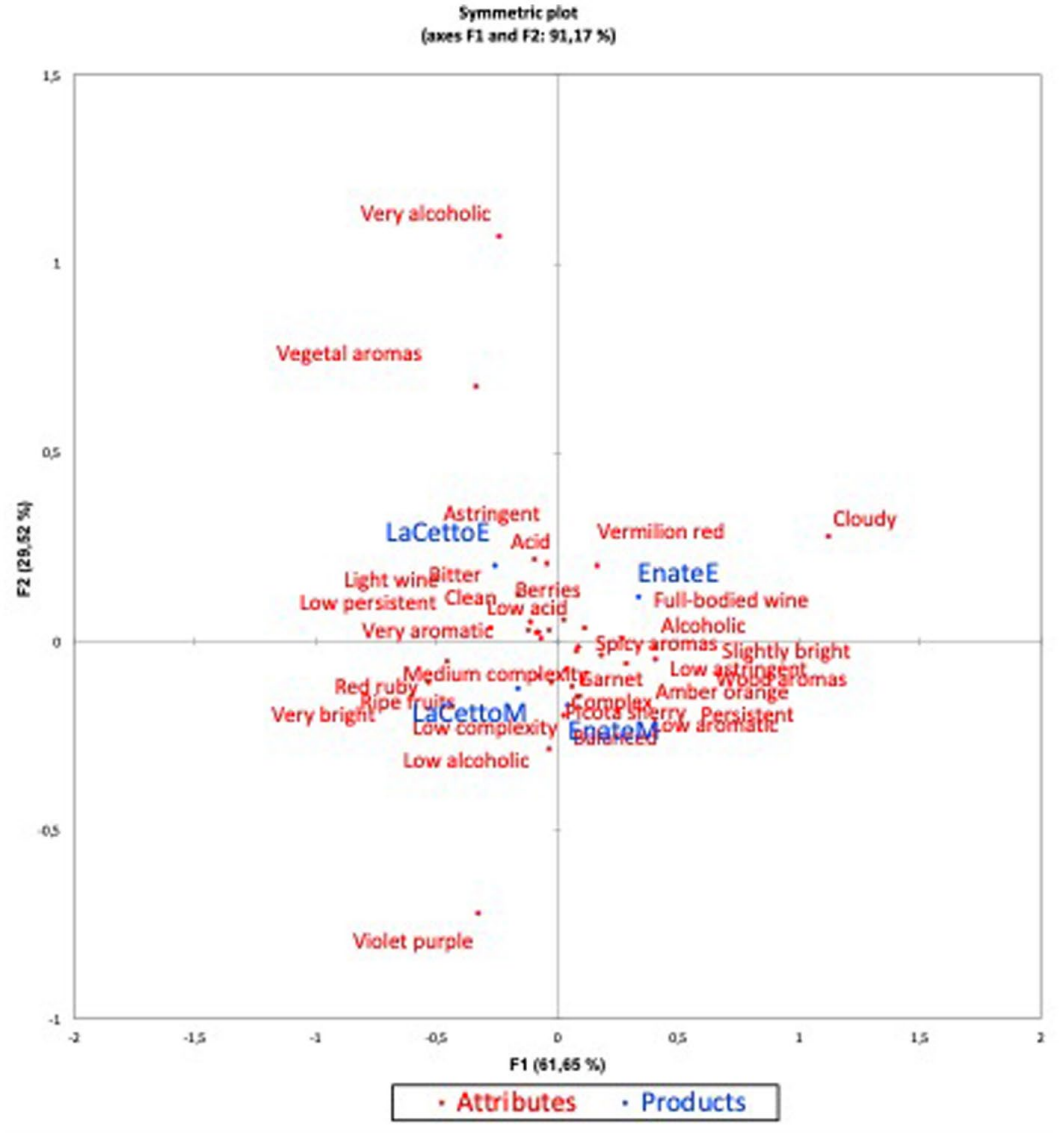
Principal coordinate analysis of red wine attributes and products. Note: F: Dimensions. Attributes: red wine attributes. Products: wine sample by country. EnateM (Enate wine tested in Mexico), EnateE (Enate wine tested in Spain), LaCettoM (L.A. Cetto wine tested in Mexico), LaCETTOE (L.A. Cetto wine tested in Spain).

In the case of red wine attributes according to country (Figure 2), the principal coordinate analysis map explained 91.17% of the variability of the wines on two axes (F1 and F2), clearly showing differences between the attributes perceived by tasters in both wines in the two countries.

For the L.A. Cetto red wine, the terms most frequently mentioned to describe the product by Spanish participants were “*Astringent*,” “*Acidic*,” “*Medium complexity*,” and “*Low alcoholic*,” while according to the perception of Mexican participants it was *“Low alcoholic*,” “*Simple complexity*,” and “*Very bright*.” The Enate red wine was perceived by Spanish participants as “*Full-bodied wine*,” “*Alcoholic,”* and “*Vermilion red*,” while Mexican participants perceived it as “*Bigarreau cherry*,” “*Woody aromas*,” and “*Balanced*,” among others.

Both Figures 1, 2 show that the wine samples were perceived in totally different ways in the two countries, a fact that is more noticeable in the red wine, due to their higher variability. Moreover, the wines are located on both sides of the axes, demonstrating in some way the discrimination of the samples. That is, they are not together because there were differences in perception between the two countries.

**Figure 3:**
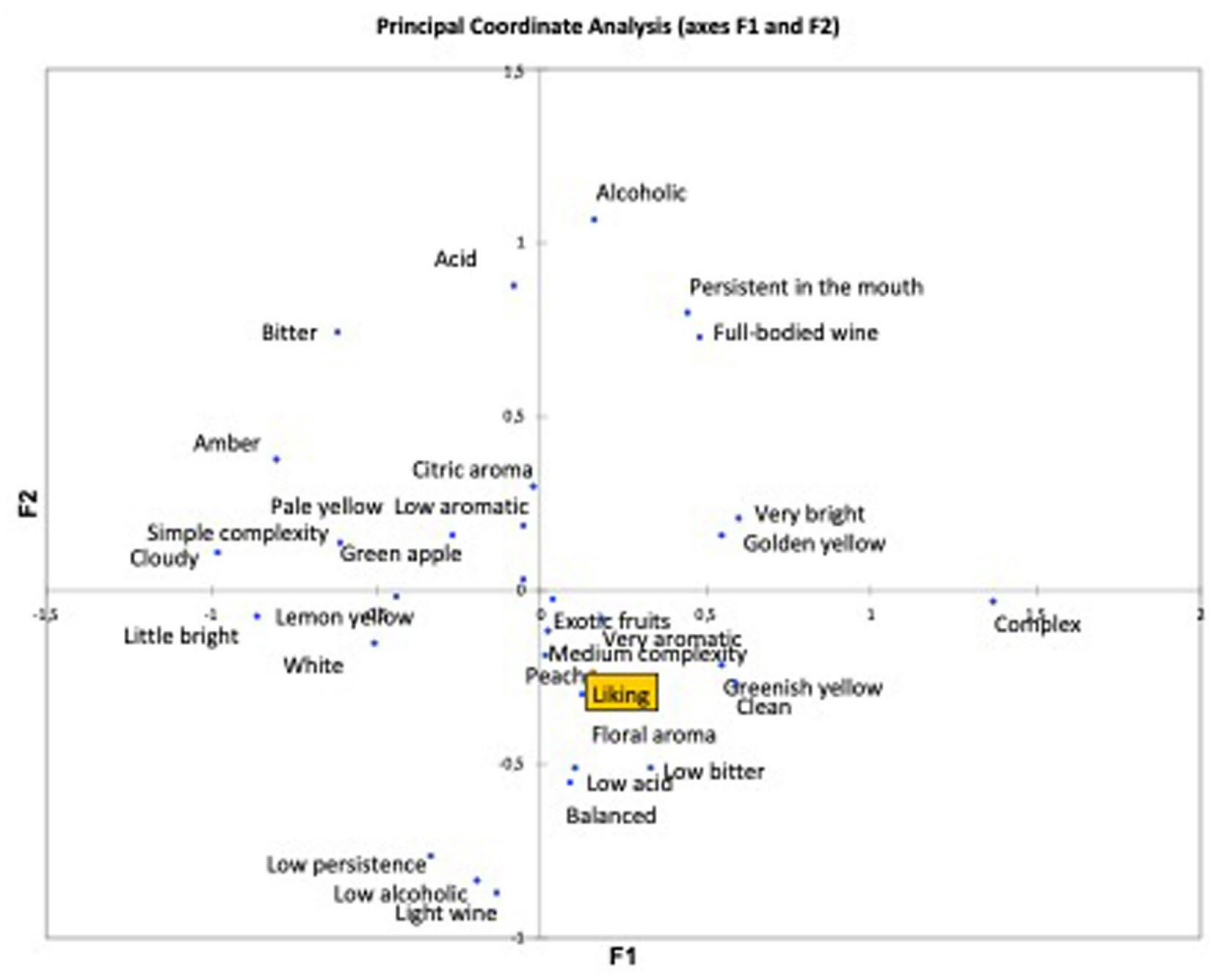
Coordinate analysis of attributes in relation to the acceptability of white wine.

**Figure 4:**
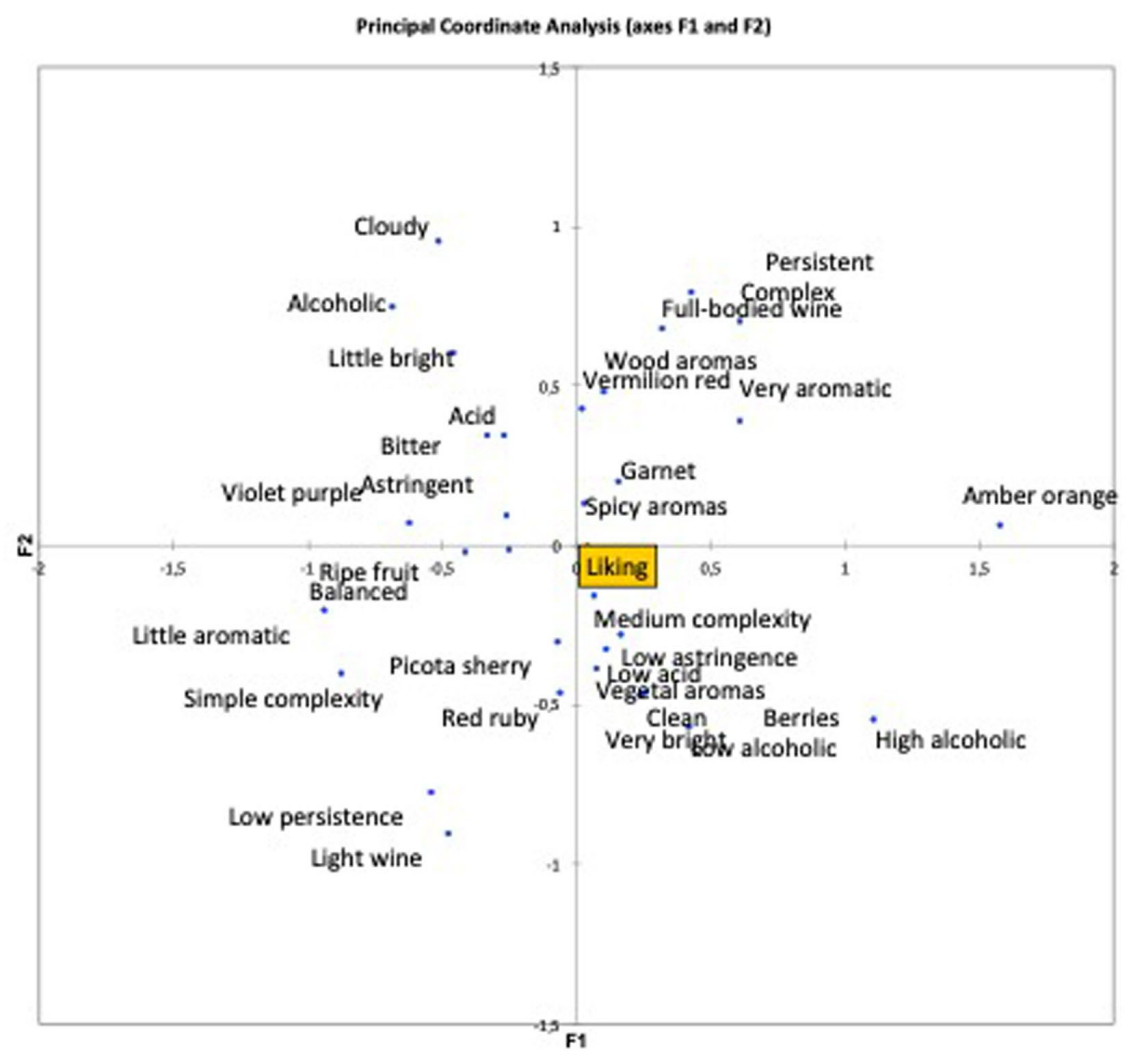
Coordinate analysis of attributes in relation to the acceptability of red wine.

Figures 3, 4 indicate the parameters that have most affected the wine’s score, which are those around *Liking* (acceptance). In other words, the aspects of the wine that most influence the acceptance of a wine and rating the product higher. In this case, the most used descriptors in relation to white wine were “*Complex*,” “*Not very bright*,” and “*Persistent in the mouth*,” among others (Figure 3).

In relation to the analysis of attribute coordinates of the acceptability of red wine, Figure 4 shows how the tasters who scored the red wine highest also chose the attributes “*Low acidity*,” “*Low astringency*,” and “*Medium complexity*,” among others, which are closest to *Liking* (Meyners et al., 2013). Although in this case, as acceptance is located in the center of the graph, there is not such a clear trend with respect to the attributes chosen, whereas in the white wine, acceptance is clearly located in the upper left quadrant dominated by certain attributes, and far from the attributes in the lower right quadrant. Here, however, acceptance is equidistant from the attributes in all directions, which means that in the case of red wine, the attributes chosen are not so decisive for acceptance.

Although only less than 40% of the analyzed attributes discriminated the wines, which were very similar, the analysis showed that the chosen lexicon is useful to describe and explain the differences between wines, countries, and their relationship with acceptability.

#### 3.2.3. Simple preference test

The results of the preferences between the two samples of red wine in the two countries are shown in Table 3. In both types of wine, there was a clear preference for Enate in both Mexico and Spain. In the case of white wine, 52.2% of Mexican participants and 67.5% of Spanish participants chose Spanish wine over Mexican wine. In the case of red wine, 70% of the Mexican population and 75% of the Spanish population preferred Spanish wine to Mexican wine.

**Table 3:** Number of tasters who preferred each sample in each country.

		Enate	L.A. Cetto	Total tasters
White wine	México	21	19	40
	Spain	27	13	40
	**Total**	**48**	**32**	**80**
Red wine	México	28	12	40
	Spain	30	10	40
	**Total**	**58**	**22**	**80**

Bold numbers is the total, the summatory of the mexican and spanish wine tasters which prefer the Enate or the L.A Cetto wine, according to White wine and red wine.

The results of the binomial test determined that in each country Enate red wine was significantly preferred (< 0.025) to L.A. Cetto, while Enate white wine was preferred only in Spain as, in the case of Mexico, there was no clear preference (> 0.025). Therefore, it could be affirmed with 95% confidence that it is not a product of chance that Enate red wine was chosen more in both populations and white wine in Spain only. The preferences in Mexico may be a product of chance or that there is no clear preference for one wine or another. On the other hand, when we gathered all the consumers together, there was a clear preference (<0.025) for Enate wines with more than 95% confidence.

After this exhaustive analysis, we can say that the wines from the Spanish winery were preferred by both study populations. The truth is that the Spanish participants showed a preference for their own wines; the panelists were more critical when it came to choosing, they are able to be more accurate because they have a wine culture rooted in everyday life that Mexico consumers lacks. the panelists were more critical when it came to choosing, they are able to be more accurate because they have a wine culture rooted in everyday life that Mexico consumers lacks. However, the Mexican population’s perception and appreciation of Spanish wines is likely due to the consumption of imported wines (70%), of which Spanish wines represent 30% of the total national consumption (Consejo Mexicano Vitivinícola, 2018).

### 3.3. Qualitative test

#### 3.3.1. Word association task

Tables 4, 5 show the terms that the Mexican and Spanish population generated to characterize the sensory profile of the wines (Pagliarini et al., 2013), which are divided into three dimensions (De Albuquerque et al., 2019) (general characteristics, sommelier, and local interpretation). According to the results obtained from the Mexican population (Table 4), the first dimension is composed of four categories (type of beverage, origin, associated attributes, and purpose), the second of two categories (service and sensory aspects) and the third of four categories (historical-social context, beliefs, related activity, and events), making 10 categories in total.

**Table 4:** Dimensions, categories, total number, and most cited terms used by the Mexican population to describe wine.

Dimensions	Categories	Total number of terms	Most cited terms
General characteristics	Type of beverage	32	Fermented beverage, fermentation, alcoholic beverage, alcohol
	Origin	15	Grape must, grape, grape must juice, France, vineyard, grape vine, grape blend
	Associated attributes	5	Fancy, calm, excellence, complex, elegant
	Purpose	4	Enjoy, taste, delight
Sommelier	Service	5	To pair, to eat, pairing
	Sensory aspects	7	Aromas, flavors, sweetness, delicious, tasty, taste
Local interpretation	Historical-social context	2	Mediterranean culture, with history
	Believes	3	Life, drink of gods
	Related category	2	To enjoy
	Events	1	Special events

**Table 5:** Dimensions, categories, total number, and most cited terms used by the Spanish population to describe wine.

Dimensions	Categories	Total number of terms	Terms
General characteristics	Type of beverage	15	Fermented beverage, fermentation, alcoholic beverage, alcohol
	Origin	7	Vineyard, grape
	Associated attributes	4	Freshness, diversity, complementary, sybarite
	Purpose	4	Tasting, enjoyment, experience
Sommelier	Service	8	Pairing, to accompany food, to eat, to enhance a meal
	Sensory aspects	6	Flavor, delicious, astringency, sweetness, aroma, taste, smell, notes, fragrances
	Gastronomy	4	Stews, broth, dinner, gastronomy
	Education	3	Dedication, knowledge, work, profession, understanding, science
Local interpretation	Historical-social context	10	Art, tradition, culture, evolution, lifestyle, territory, Romans, triclinium, expression, Dionysus
	Related category	15	Fun, relaxation, sharing, leisure, drunkenness, living, resting, laughing, joy
	Events	6	Festivity, celebration
	Hedonism	4	Pleasure, gustatory pleasure, “hedoné”

In Mexico, the words most associated with wine were mostly in the subcategory of “*Type of beverage*” (32), followed by “*Origin*” (15) and “*Sensory attributes*” (7). Among the most frequently mentioned terms were “*Alcoholic beverage*,” “*Fermentation*,” “*Fermented beverage*,” “*Grape*,” “*Grape must*,” and “*Flavor*.”

Table 5 shows the terms that the Spanish population used to characterize wine, also divided into three dimensions (De Albuquerque et al., 2019) (general characteristics, sommelier, and local interpretation). According to the results obtained from the Spanish population, the first dimension is composed of four categories (type of beverage, origin, associated attributes, and purpose), the second of four categories (service, sensory aspects, gastronomy, and education) and the third of four categories (historical-social context, related activity, events, and hedonism), making 12 categories in total.

However, in Spain, the most frequently used terms were equally distributed among the subcategories of “*Type of beverage*” (15) and “*Related activity*” (15), and terms from the subcategory “*Social-historical context*” were also mentioned to a large extent (10). Among the most commonly used terms in these three subcategories we found “*Pairing*,” “*Sharing*,” “*Fermented beverage*,” “*Tradition*,” and “*Culture*,” among other words.

Therefore, we could mention that the participants of the cross-cultural study evaluated wine from different points of view: Mexican participants focused more on defending the very concept of wine, while Spanish participants showed much more familiarity (Bryant et al., 2019) with the product due to the higher consumption of wine as a country (OIV, 2021) and the regular exposure to this product, which is very much a part of everyday life. However, in Mexico there is no culture of drinking wine habitually (ICEX, 2018) in addition to the fact that the wine industry in this country has faced difficulties throughout history and has not been established as part of its culture (Hidalgo Togores and Hidalgo Fernández-Cano, 2011). The richer language and number of expressions used by Spanish participants compared with Mexican participants is noticeable (Tables 4, 5). Another remarkable thing expressed by the association task is that the Mexican participants attributed the wine to other cultures (e.g., France) while the Spanish participants attributed it to their own culture.

This study, in which most of the participants were students, demonstrates that the approach to the knowledge of wine of the two different cultures can be successfully investigated using the combination of the simple preference test, acceptance test, CATA test, and others like projective mapping combined with qualitative tests like word association tasks or list descriptors of the product. For future research, it is important to focus on the selection of consumers that best represent the real population and how to perform the different tests to avoid bias due to the interference between the tests performed.

## 4. Conclusion

This study has shown that there are significant differences in the perception and preference of wine in Mexico and Spain due to the fact that they are evaluated through a cultural lens. The results obtained from both populations in all the tests performed have been concurrent and were related to each other to determine the perception of wine in the same population.

The population that is most exposed to the product, in this case the Spanish population, associates wine as a way of life (both in food and in daily activities), tradition, and knowledge due to familiarity, which is largely determined by geographical location. This interacts with specific experiences in the wine field (i.e. wine knowledge) and habitual consumption, so a certain requirement has been observed during the sensory evaluation, both in hedonic and descriptive tests. In contrast, the Mexican population does not consider wine as part of their traditional culture; thus, their point of view is more objective and they are more specific in their definitions. Furthermore, they not emitted a solid evaluation than the Spanish; they did not have a determining criterion when evaluating the wines and there was no clear difference in preference, especially in the case of the white wine. Overall, they were not as pundits as the Spanish when it came to awarding a score or attribute.

In relation to the wines, those from Spain were better appreciated both in terms of perception and preference, so were succeed in both countries as expected. However, although the Enate wines were better rated, their scores were not very high (below 6.5), and a higher acceptance was expected. Similarly, with CATA, less significant attributes than expected were attributed to both wines, probably because wines of similar quality were chosen, but it was found that Enate and L.A. Cetto wines were perceived organoleptically differently.

Cross-cultural research involving wine and wine tasting is in its infancy; it is beginning to provide evidence of how wine is appreciated. This study generates new quantitative and qualitative research data from which sensory science and the wine industry will benefit. As was mentioned, sensory analysis techniques are useful tools to understand how wine culture is built and its relationship with the products as demonstrated in the present study.

## Data availability statement

The raw data supporting the conclusions of this article will be made available by the authors, without undue reservation.

## Author contributions

IO, ES, MR, and AS contributed to the conception and design of the study. IO and AS contributed to the sensory analyses and data collection in Mexico and Spain. MR contributed to the statistical analyses. IO and ES contributed to writing this manuscript. All authors contributed to the article and approved the submitted version.

## Conflict of interest

The authors declare that the research was conducted in the absence of any commercial or financial relationships that could be construed as a potential conflict of interest.

## Publisher’s note

All claims expressed in this article are solely those of the authors and do not necessarily represent those of their affiliated organizations, or those of the publisher, the editors and the reviewers. Any product that may be evaluated in this article, or claim that may be made by its manufacturer, is not guaranteed or endorsed by the publisher.

## Footnotes

^1^ https://www.r-project.org/
